# Immunoliposomes with Simvastatin as a Potential Therapeutic in Treatment of Breast Cancer Cells Overexpressing HER2—An In Vitro Study

**DOI:** 10.3390/cancers10110418

**Published:** 2018-11-01

**Authors:** Lucyna Matusewicz, Joanna Podkalicka, Aleksander F. Sikorski

**Affiliations:** Department of Cytobiochemistry, Faculty of Biotechnology, University of Wroclaw, ul. F. Joliot-Curie 14a, 50-383 Wrocław, Poland; matusewicz.lucyna@gmail.com (L.M.); joanna.szymak@gmail.com (J.P.)

**Keywords:** simvastatin, immunoliposomes, HER2-overexpressing breast cancers

## Abstract

Lipophilic statins are promising candidates for breast cancer treatment. However, anticancer therapy requires much higher doses of statins than can be delivered orally, and such high doses are known to exert more adverse effects. The main objective of our study was to design a targeted, therapeutic liposomal carrier of simvastatin characterised by high stability and specificity towards breast cancer cells. We chose SKBR3, the cell line that showed the highest sensitivity for simvastatin and liposomal simvastatin treatment. Additionally, SKBR3 has a notably high expression level of human epidermal growth factor receptor 2 (HER2), which we used as a target for our immunoliposomes. To do so we attached humanized anti-HER2 antibody to the envelope of liposomes. We tested the stability and selectivity of the proposed formulation along with the toxicity, ability to induce apoptosis and the effect on signalling pathways involving Akt and Erk kinases. The immunoliposomal formulation of simvastatin is characterized by long-term stability, high selectivity towards HER2-overexpressing breast cancer cells, low non-specific cytotoxicity and effective inhibition of the growth of target cells, presumably by inhibition of signalling pathways and induction of apoptosis. Hence, for the first time, we propose the use of immunoliposomes with simvastatin, targeted directly towards breast cancer cells overexpressing HER2. The prepared immunoliposomes may become a proof of concept in developing new anticancer therapy.

## 1. Introduction

According to the American Cancer Society regarding data from 2017, breast cancer is the second (after lung cancer) leading cause of cancer death among women. Breast cancer encompasses several distinct subtypes that differ in terms of molecular characteristics, responses to adjuvant therapies and clinical outcomes [1]. As sensitivity of particular cancer types to various agents (cytostatics, inhibitors or antibodies) markedly varies, it is important to characterize the molecular features in order to choose the most suitable target for the therapy [2].

A promising target for the treatment of one of the subtypes of breast cancer is human epidermal growth factor receptor 2 (HER2). HER2/neu (or ErbB2) is an orphan receptor that belongs to the family of epidermal growth factor receptors (EGFRs). Its overexpression was noted in 20–25% of breast tumours and is associated with an aggressive form of the disease with poor patient prognosis [3]. Trastuzumab (Herceptin) is a humanized monoclonal antibody which binds to the ectodomain of HER2 [4] and was the first HER2-targeted agent approved by the US Food and Drug Administration (in 1998) for clinical use in the breast cancer patients. Trastuzumab is frequently used in combination with standard chemotherapy [5].

Despite all that we know about breast cancer and the tremendous progress in its treatment, there is still a great need to improve existing therapies. One promising strategy in this field is the use of liposomes as drug delivery systems. By entrapping the drug, liposomes change its pharmacokinetics, usually by significantly prolonging its half-life and markedly influencing biodistribution of the drug. Since their first description in 1965 by Sir Alec Bangham [6,7], the structure and size of liposomes have been modified many times to achieve drug carriers with the best properties. Nowadays, large unilamellar liposomes (LUVs) with a diameter around 100 nm are the most often investigated as potential drug carriers. Liposomes with cholesterol or sphingomyelin incorporated into their bilayer are known to have reduced leakage of their content in comparison to liposomes made only of glycerol phospholipids. Modifying the surface of liposomes with polyethylene glycol (PEG) importantly reduces the rapid clearance of liposomes from the circulation by the cells of the mononuclear phagocyte system (MPS); hence pegylated liposomes (stealth liposomes) stay in the circulation much longer than non-pegylated ones [8,9,10]. Most importantly, it is possible to conjugate complete or active fragments of antibodies to the surface of liposomes to obtain so-called immunoliposomes. Immunoliposomes are thought to selectively deliver a drug to the desired site in the body as antibodies selectively recognize and bind to the specific antigen on the surface of target cells, thus reducing possible side effects (for a review see [11]). It is worth emphasising that the therapeutic advantage of immunoliposomes over non-targeted liposomes stems not only from the higher amount of drug molecules that reach the target cell, but also from the fact that cells actively take up liposomes by endocytosis [8].

The role of statins in almost all types of cancers has been discussed for about three decades. In the early 1990s, statins, cholesterol-depleting drugs commonly used in a treatment of cardiovascular diseases, were shown to also have anticancer properties. The main obstacle preventing use of this class of drugs in anticancer therapy is that they need to be supplied in relatively high doses, even 500 times higher than those used in the treatment of hyperlipidemic syndromes. Such high doses may lead to many serious side effects, including myopathy and rhabdomyolysis [12]. Moreover, it is not possible to obtain such a high statin concentration in the circulation when taken orally, because of their pharmacokinetic properties. Statins possess short elimination half-lives (mostly 3 h or less) and except for pravastatin all of them are extensively bound to plasma proteins, which makes them pharmacologically inactive. Most statins have low systemic bioavailability (below 20%) [13] as a result of extensive hepatic first-pass metabolism. To overcome these obstacles, many strategies were taken into consideration. One of the most promising was to deliver statins as proliposomes [14] or liposomes [15,16,17,18,19].

Regarding all the difficulties mentioned above, the main aim of this study was to design liposomes with simvastatin incorporated into the liposome bilayer, selectively targeted towards breast cancer cells overexpressing HER2. Among all statins we chose simvastatin because of its potent antitumor activity and particularly low bioavailability (around 5%). The stability, selectivity and efficacy of immunoliposomes were tested. We assumed that the drug in its immunoliposomal form will accumulate selectively in breast cancer cells overexpressing HER2, so its concentration will be high only in tumours, avoiding an overall systemic response and toxic side effects. The efficacy of our simvastatin immunoliposomes was compared to non-targeted liposomes and the drug in its free form.

## 2. Results

Our aim was to construct simvastatin-loaded liposomes targeted with a specific antibody in order to deliver relatively high doses of drug to specific tumours in the body. At first, we constructed liposomes with simvastatin, and tested their physicochemical properties. We also chose a cell line among breast cancer cells of different molecular characteristics that showed the highest sensitivity for simvastatin treatment along with high expression of antigens, which we could use as a target for immunoliposomes. After that, we checked the effect of immunoliposomes on the biology of the selected cell line.

### 2.1. Design of Simvastatin Liposomes: Cholesterol as a Key Factor Limiting the Capacity and Toxicity of Liposomes

The first stage of this study was to construct a non-targeted liposomal formulation of simvastatin. The liposomal formulations which were tested are shown in Table 1. The criteria of the selection of the best formulation were, first of all, the efficiency of encapsulation, secondly, the stability of formulation, which was initially addressed by checking the time after which simvastatin crystals appear in the liposomal suspension during its storage at 4 °C and also nonspecific toxicity of low cholesterol content liposomes on certain cell lines (see below).

As a starting point the composition of our liposomes was: hydrogenated soya bean phosphatidylcholine (HSPC), cholesterol (chol), 1,2-distearoyl-sn-glycero-phosphoethanolamine-N- (poly (ethylene glycol) 2000) (DSPE-PEG) and simvastatin (SIM) in 45:40:5:10 molar ratio. However, in this type of formulation, simvastatin precipitated out within 1–2 days.

Simvastatin is a highly lipophilic drug, so it should be easily incorporated into the liposome bilayer in a passive way. Its structure contains a main polyketide part and the hydroxy-hexahydro naphthalene ring system, to which side chains are attached at positions C8 and C6. Because of the structure, we hypothesised that simvastatin could successfully replace cholesterol rather than HSPC in the initially proposed liposome bilayer. There are several attempts of cholesterol-free liposome constructions for delivery of hydrophobic drugs (e.g., [20,21]). Moreover, as simvastatin is an inhibitor of cholesterol synthesis, our goal was to minimize cholesterol content in liposome bilayer not to deliver it along with the drug.

In the next trial we decided to increase the amount of HSPC and decrease cholesterol content to the level which does not affect the stability of liposomal simvastatin formulation. We achieved our goal with 80.65 mol % of HSPC and 5.35 mol % of DSPE-PEG, which together constituted 86 mol % of the liposomal bilayer. The remaining 14 mol % constituted cholesterol and simvastatin, and we varied their ratio to obtain liposomes that were the most stable and efficient in terms of drug encapsulation. At first, we tested formulations with an equal molar ratio of simvastatin (SIM) and cholesterol (1:1) and a simple formulation which did not contain cholesterol but contained 14 mol % of simvastatin. Liposomes without cholesterol were stable longer than those with a 1:1 chol:SIM ratio, but their drug encapsulation efficiency was lower by about 10%. Next, we tested liposomal formulations with a 0.5:1 chol:SIM molar ratio. These liposomes were stable over 1 month and had encapsulation efficiency comparable with liposomes with a 1:1 chol:SIM molar ratio. Finally, we tested a formulation in which 20% of HSPC was replaced with 1,2-distearoyl-sn-glycero-3-phosphocholine (DSPC). The last liposomal formulation was stable for the longest time period, equalling about 8 months, as we did not observe precipitation of simvastatin from liposomes, and was also characterised by the highest encapsulation efficiency (EE) (Table 1).

In conclusion, we observed that the most favourable chol:SIM molar ratio in the liposomal envelope is 0.5:1 (Table 1, formulations No. 3 and 4). Also, we noted that increases of the content of phosphocholine (PC) consisting of long, saturated, fatty acyl chains in the liposomal envelope stabilizes incorporated simvastatin and inhibits its precipitation (see stability and EE of formulation No. 4 in Table 1).

One of the following steps aiming to determine the optimal lipid composition of the designed liposomes was to check the viability of selected breast cancer cells after treatment with the empty liposomal envelope. The compositions of tested empty liposomes were almost identical to the composition of the drug-carrier preparation, with the difference that in one case 9.5 mol % of simvastatin was replaced with HSPC, and in the second with cholesterol (see Table 2).

**Figure 1:**
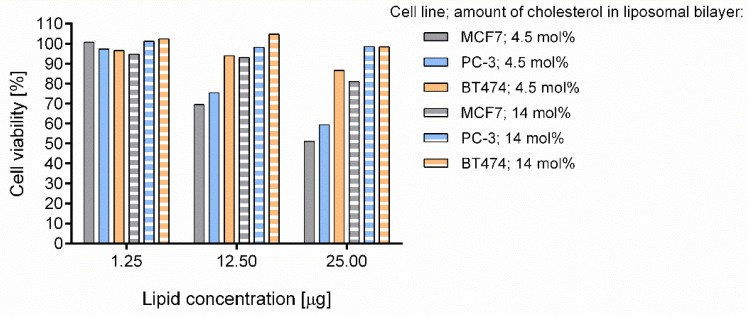
Viability of breast and prostate cancer cell lines after treatment with empty liposomes. Cells were treated with empty liposomes at concentrations corresponding to 1, 10 and 20 µM of simvastatin liposomes for 48 h. Viability of cells was assessed by MTT (3-(4,5-dimethylthiazol-2-yl)-2,5-diphenyltetrazolium bromide) tetrazolium) reduction assay.

Interestingly, we observed that if we reduced the amount of cholesterol in the liposomal envelope to values as low as 4.5 mol %, the toxicity of empty liposomes increased significantly for some of the cell lines tested (we tested breast cancer cell lines MCF7 and BT474). Apart from breast cancer cells, we tested the toxicity of empty liposomes with a low cholesterol level on prostate cancer cell line PC-3. The viability of this cell line is highly dependent on cholesterol level. It was found that empty liposomes with low cholesterol decreased viability of prostate cancer cells to 60% (Figure 1). Cholesterol exchanges relatively freely between cells and liposomes and in our opinion this effect caused decreased viability of cells of some lines which were more sensitive to cholesterol depletion.

Based on the obtained results, in all subsequent experiments control liposomes of 14 mol % cholesterol were used in order to maintain the empty liposomes non-toxic for the cells while simvastatin content in the lipid membrane was 9.5 mol %.

However, the main aim of our project was to develop targeted statin therapy. To achieve targeted delivery of simvastatin we planned to use specific, commercially available antibodies; thus the crucial issue was the attachment of antibodies to the liposome bilayer. The method of antibody attachment to the liposomes was previously successfully used in our laboratory [22]. For simvastatin liposomes we chose trastuzumab (Herceptin), a humanized antibody that recognizes the HER2/neu receptor as a target molecule.

### 2.2. Characterisation of Non-Targeted Liposomes and Immunoliposomes Containing Simvastatin

To characterise both immunoliposomes and non-targeted liposomes containing simvastatin as well as their empty counterparts, we measured the diameter, polydispersity index (PDI) and zeta potential of all formulations. For drug carriers we also calculated EE and drug loading (DL) values and for immunoliposomes we measured the amount of antibody that was successfully attached to the envelope.

The average diameter of non-targeted liposomes was around 120 nm and the average size of immunoliposomes was slightly larger, around 125 nm, which is probably due to the presence of antibodies on the surface of the liposomes. Only liposomes with a PDI lower than 0.1, which proves the high homogeneity of the obtained formulations, were used in further experiments. The zeta potentials of all liposomal formulations were negative. The detailed values are presented in Table 3.

To obtain more detailed characteristics, we also determined the concentration of antibodies attached to the immunoliposomal formulations. On average, we measured 45.8 µg of conjugated IgGs per 1 mg of phospholipids in immunoliposomal formulations. This result is in the range of antibody concentrations used by other research groups to design targeted liposomes [9,23]. Such a low concentration of attached antibodies should prevent toxicity caused by antibodies themselves, but should be sufficient to enable binding to targeted cells.

Finally, to determine the efficacy of simvastatin incorporation into the liposome bilayer, we calculated EE and DL values, according to equations presented in the Materials and Methods section (Section 4.6). For non-targeted liposomes with simvastatin EE = 63%, DL = 6.8%; for simvastatin immunoliposomes EE = 65% and DL = 6.8%. DL values are typical for liposomes obtained via the lipid film hydration method, as when using this method, it is very hard to obtain DL values higher than 8–9%. EE values around 65% may be considered as low, however it should be noted, that simvastatin is a highly hydrophobic drug which is incorporated into liposomal bilayer and does not accumulate inside liposomal vesicle. Additionally, the size of our liposomes was of around 120 nm which facilitates circulation in the body, but on the other hand limits liposomes capacity. It should be noted that the EE values obtained for our liposomes are higher than those determined for other simvastatin liposomes of similar size. It is also worth noting that the attachment of antibodies does not reduce the amount of simvastatin in the liposome bilayer.

### 2.3. Stability of Designed Liposomes and Immunoliposomes

**Figure 2:**
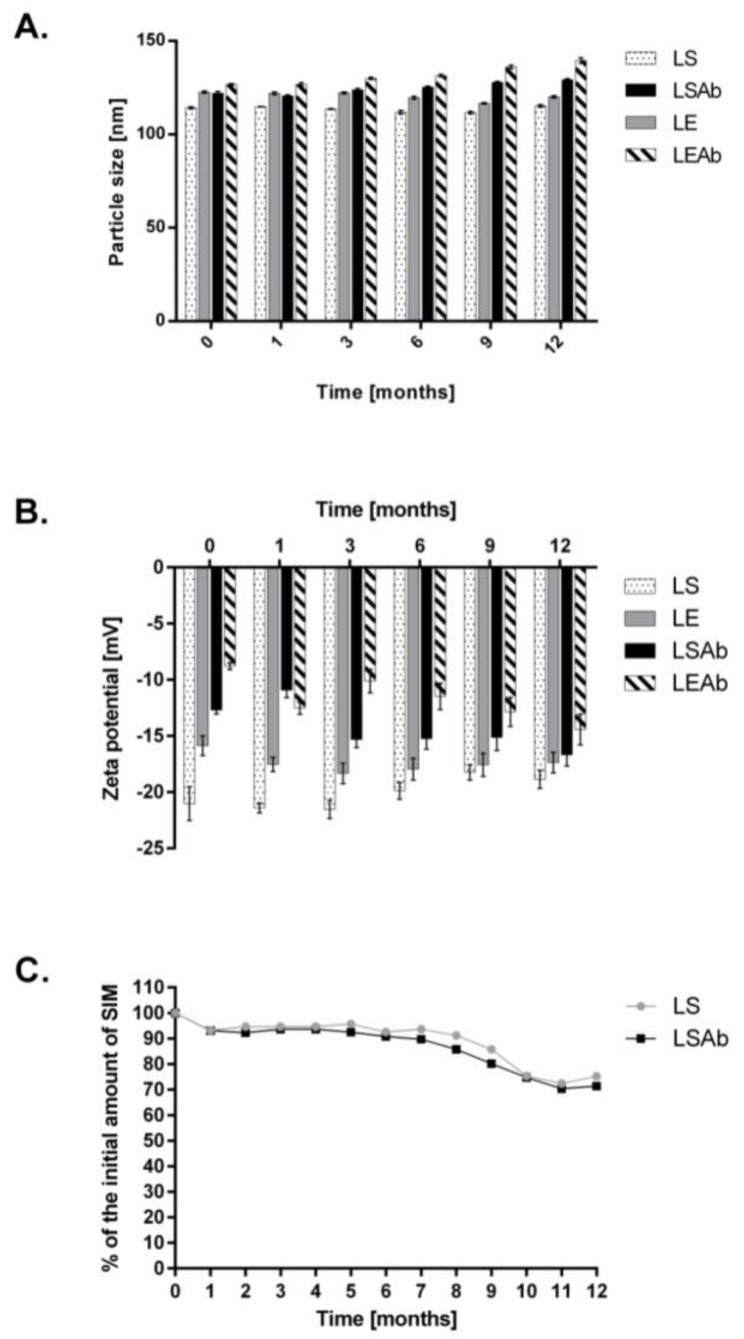
Stability of targeted and non-targeted liposomes during 12 months of storage as a suspension. Both liposomal and immunoliposomal formulations of simvastatin were stored at 4 °C. After every month, changes in liposome diameter (**A**), zeta potential (**B**) and simvastatin content (**C**) were measured. LS—non-targeted liposomes with simvastatin, LSAb—immunoliposomes with simvastatin, LE—non-targeted empty liposomes, LEAb—empty immunoliposomes.

In order to study the stability of simvastatin immunoliposomes, we performed two experiments. In the first test, liposomes were stored as a suspension at 4 °C for over 1 year. Every 30 days the diameter, zeta potential and simvastatin content in liposomes and immunoliposomes were measured. The diameter of both drug-containing liposomal preparations and their empty counterparts did not change significantly during storage. Changes in diameter of non-targeted liposomes were less than 5 nm while maximal changes in diameter of immunoliposomes were around 10 nm (Figure 2A). There were only minor changes in zeta potentials of all formulations (Figure 2B). After 6 months of storage an MTT (3-(4,5-dimethylthiazol-2-yl)-2,5-diphenyltetrazolium bromide) tetrazolium) reduction assay was performed to check in vitro effectiveness of all preparations and the results were comparable to those obtained for freshly prepared liposomes [24]. The drug content decreased to 90% of the initial content after 1 month of storage but then simvastatin concentration in immunoliposomes was essentially stable for at least 8 months with 85% of initial simvastatin content still observed (Figure 2C).

**Figure 3:**
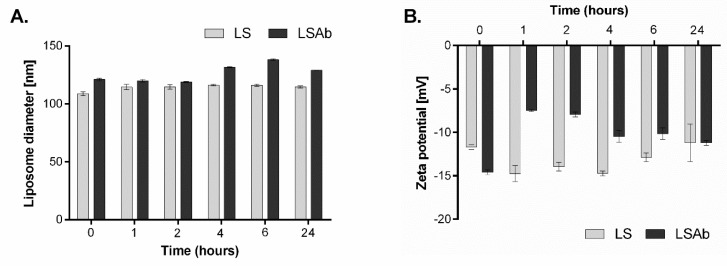
Stability of simvastatin liposomes and immunoliposomes upon incubation with human plasma. Simvastatin liposomes and immunoliposomes were incubated with 50% human plasma at 37 °C. After indicated time period, changes in liposomes diameter (**A**) and zeta potential (**B**) were measured.

In another series of experiments, the stability of simvastatin immunoliposomes upon incubation with human plasma was determined. The experiments were performed as described in Materials and Methods (Section 4.6). After 6 h incubation of liposomes with 50% human plasma, the diameter of immunoliposomes increased from 120 nm to around 140 nm, but quite surprisingly, after 24 h it returned to its initial size (Figure 3A). Zeta potential of immunoliposomes increased after 1 h incubation with human plasma to almost half of its initial value, but then gradually decreased to reach a negative value close to the initial one (Figure 3B).

Simvastatin immunoliposomes, like simvastatin non-targeted liposomes, are stable over 8 months when stored as a suspension at 4 °C (Figure 2). Despite small changes in their size and zeta potential, simvastatin immunoliposomes were also stable in the presence of 50% human plasma for at least 24 h, which makes them potential candidates for in vivo treatment.

### 2.4. Breast Cancer Cell Lines Overexpressing EGFR Are Sensitive to Treatment with Liposomal and Immunoliposomal Forms of Simvastatin

In order to find the best molecular target for simvastatin therapy, we determined the expression levels of receptors from the EGFR family in lysates from breast cancer cell lines of different molecular characteristics. More precisely, expression levels of EGFR and HER2 in the basal-like MDA MB 231 cancer cell line, HER2 enriched SKBR3 cancer cell line, luminal A MCF7 cancer cell line and luminal B BT474 cancer cell line were determined by Western blot analysis.

**Figure 4:**
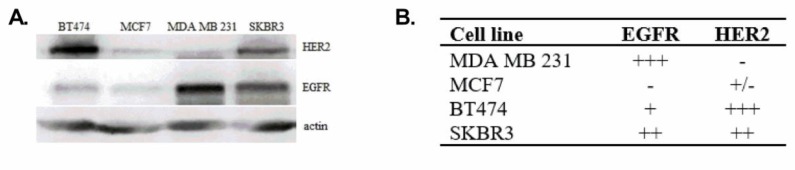
Level of HER2 (ErbB2) and EGFR (ErbB1/HER1) in different breast cancer cell lines used in the study. Expression levels of both receptors in cell lysates prepared from 2.5 × 10^5^ cells were determined by Western blot analysis (**A**). Graphical presentation of the levels of HER2 and EGFR in breast cancer cell lines (**B**) was prepared on the basis of (**A**). +: high; −: low.

The level of both receptors, EGFR and HER2, was nearly undetectable in the MCF7 cell line. A high HER2 content was found in BT474 and SKBR3 cell lines whereas in MDA MB 231 the level of HER2 was undetectable. In contrast, the EGFR level was the highest in the MDA MB 231 cell line, slightly lower in SKBR3 and very low in the BT474 cell line (Figure 4). These results are in accordance with findings of other research groups.

To examine in vitro effectiveness of designed simvastatin liposomes, all four breast cancer cell lines were treated with both liposome formulations and with the free form of the drug at the same concentration range (0.25 µM–200 µM) for 48 h. The viability of cells was assessed by MTT assay. Among selected breast cancer cell lines, the highest response to free simvastatin was observed for cell lines that overexpress EGFR, MDA MB 231 and SKBR3 (Table 4). The highest sensitivity to treatment with simvastatin liposomes was obtained for the SKBR3 breast cancer cell line (the lowest IC_50_ value, see Table 4). A significant decrease of IC_50_ value after liposomal simvastatin treatment (in comparison to IC_50_ for the free drug) was also observed for another cell line overexpressing HER2, BT474. Therefore, we decided to attach an antibody to liposomes that would specifically recognize HER2. In this case, we chose an antibody that is commercially available and commonly used in anti-cancer therapies, trastuzumab (Herceptin). The efficacy of simvastatin immunoliposomes was tested on cell lines overexpressing HER2 and, as a control, on cells that do not express the receptor. It turned out that for both breast cancer cell lines overexpressing HER2, the IC_50_ value of simvastatin immunoliposomes was lower than that for the free drug and/or non-targeted liposomes (Table 4).

Although the results for the BT474 cell line seemed to be the most attractive, as the difference between IC_50_ values for the free drug, non-targeted liposomes and immunoliposomes is the greatest, it is difficult to induce a tumour with this breast cancer cell line in vivo. BT474 cells express the oestrogen receptor, so they require additional oestrogen supplementation after implantation in mice. Keeping in mind that in the future we would like to study the effect of our simvastatin immunoliposomes in vivo, for the first time preferably on the simplest model, and taking into consideration results from the MTT assay, we decided to choose the SKBR3 breast cancer cell line as a target for our further investigation of simvastatin liposomes to which antibodies directed against the HER-2 receptor were attached. SKBR3 represents both high simvastatin sensitivity and relatively high HER2 expression, and this cell line seems an excellent model for testing immunoliposomal simvastatin formulation.

**Figure 5:**
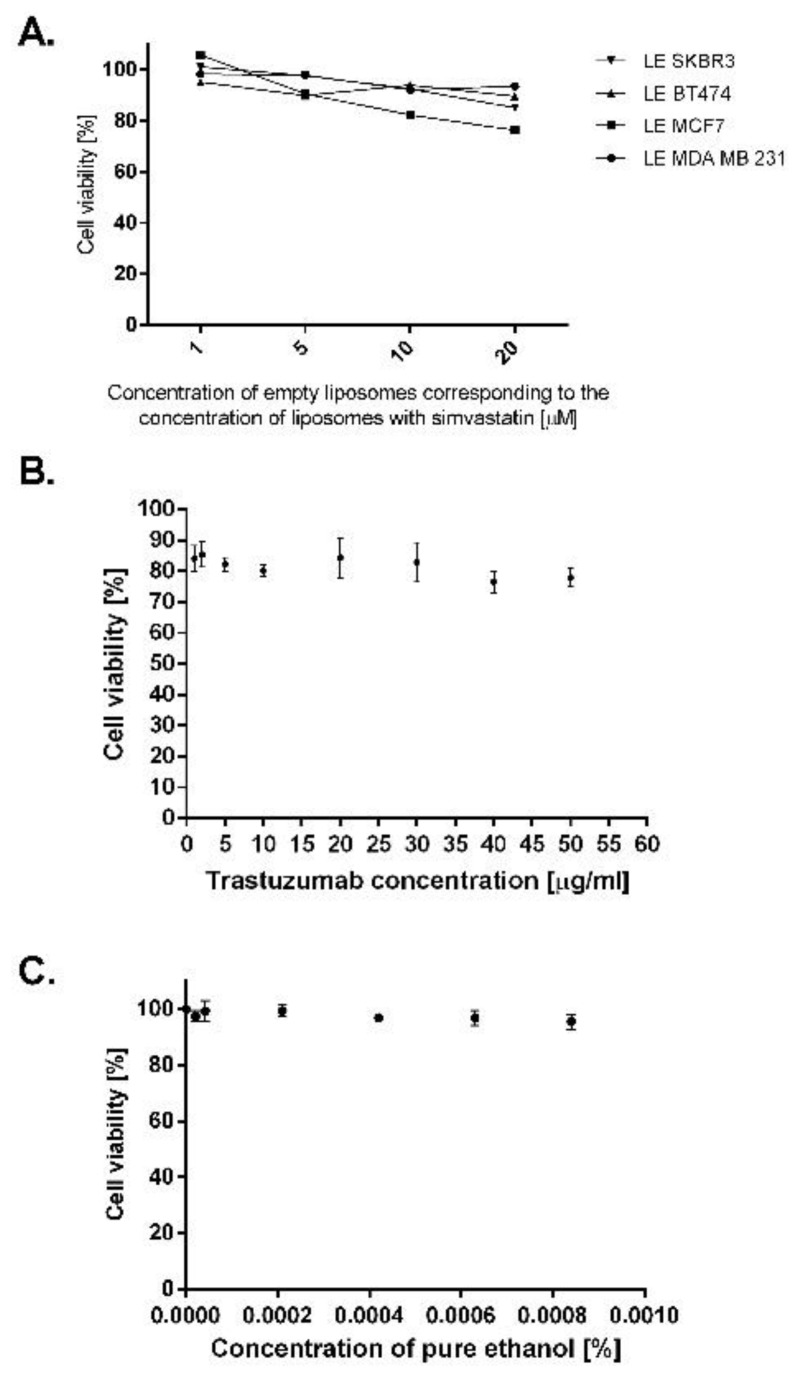
Toxicity of empty liposomal envelope towards tested breast cancer cell lines (**A**), toxicity of pure antibody (**B**) and toxicity of ethanol towards SKBR3 breast cancer cell line (**C**). Cells were treated with empty liposomes (**A**) and SKBR3 cells solely were treated with trastuzumab (**B**) or ethanol (**C**); viability of the cells was assessed by MTT assay.

Non-targeted empty liposomes and empty immunoliposomes were also tested to investigate their effect on viability of breast cancer cells. Neither non-targeted empty liposomes (Figure 5A) nor immunoliposomes used in concentrations equivalent to those delivered with immunoliposomal simvastatin were toxic for the chosen cancer cells. We also evaluated whether pure antibody or pure ethanol which was used to solubilize free simvastatin may affect the growth of SKBR3 breast cancer cells. After 48 h incubation of cells with trastuzumab at different concentrations, we observed a reduction in cell viability, which for the highest concentration of pure antibody, 50 µg/mL, was equal to 20%. We did not observe any changes in SKBR3 cells viability after 48 h of incubation with ethanol at concentrations used to deliver free simvastatin.

### 2.5. Simvastatin Immunoliposomes Are Selective towards Breast Cancer Cells Overexpressing HER2

**Figure 6:**
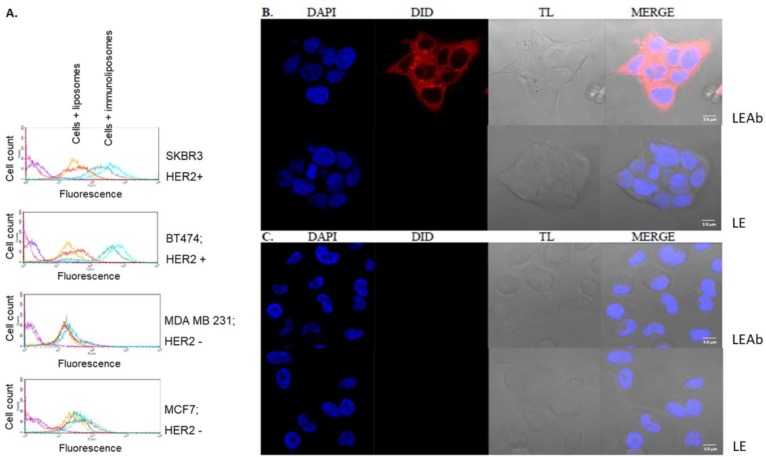
Selectivity of immunoliposomes interactions with different breast cancer cell lines. (**A**) Histograms for BT474, SKBR3, MCF7 and MDA MB 231 cells that were incubated with DiD(1,1’-dioctadecyl-3,3,3’,3’-tetramethylindodicarbocyanine)-labelled non-targeted liposomes or with DiD-labelled immunoliposomes for 30 min and 2 h. For BT474 and SKBR3 cell lines there is a significant shift to the right of the signal that is correlated with cells treated with DiD-labelled immunoliposomes (green line—30 min treatment, blue line—2 h treatment) in comparison to the signal obtained for cells treated with DiD-labelled non-targeted liposomes (yellow line—30 min, red line—2 h treatment). The shift of the signal was not observed for control cell lines. (**B**) Exemplary confocal images of BT474 breast cancer cells after 30 min of incubation with DiD-labelled immunoliposomes (top) and DiD-labelled non-targeted liposomes (bottom). (**C**) Exemplary confocal images of MDA MB 231 breast cancer cells after 30 min of incubation with DiD-labelled immunoliposomes (top) and DiD-labelled liposomes without antibodies (bottom). TL—transmitted light. Scale bar: 10 μm. Blue: nuclei stained with DAPI; Red: DiD-labelled immunoliposomes.

The selectivity of simvastatin immunoliposomes was confirmed on breast cancer cell lines which differ in terms of HER2 expression: BT474, SKBR3 (high expression of HER2) and MCF7, MDA MB 231 (low expression of HER2), using two independent methods, flow cytometry (Figure 6A) and confocal microscopy (Figure 6B). In both experiments, targeted and non-targeted liposomal formulations were labelled with 0.1 mol % 1,1′-dioctadecyl-3,3,3′,3′-tetramethylindodicarbocyanine (DiD).

The results from flow cytometry are shown in Figure 6A, where single parameter histograms associated with the FL4-H signal from DiD-labelled immunoliposomes and DiD-labelled non-targeted liposomes in each cell line are depicted. In the case of SKBR3 and BT474 cell lines treated with immunoliposomes the fluorescence histogram is substantially shifted to the right, meaning a much more intense signal compared to the histograms obtained for non-targeted liposomes. The shift is observed already after 30 min of incubation and remains at the same level after 2 h. On the other hand, for cells that do not express HER2, the histograms obtained for both targeted and non-targeted liposomes are at the same level and remain overlapped even after 2 h of incubation with immunoliposomes. These observations suggest high specificity of immunoliposomes–cell surface interactions, which was further confirmed by confocal microscopy.

Confocal microscopy clearly showed that 30 min of incubation is enough for DiD-labelled immunoliposomes to bind efficiently to the surface of BT474 cells overexpressing HER2 (Figure 6B, top), but is not sufficient for non-targeted liposomes (Figure 6B, bottom). After 2 h of incubation still no signal from non-targeted liposomes was observed [25]. Similarly, binding of neither HER2-targeted immunoliposomes (Figure 6C, top) nor non-targeted liposomes (Figure 6C, bottom) to the MDA MB 231 cells that do not express HER2 was observed.

The obtained results indicate high selectivity of immunoliposomes towards target cells that overexpress HER2.

### 2.6. Immunoliposomal Simvastatin Induces Apoptosis in SKBR3 Cells

**Figure 7:**
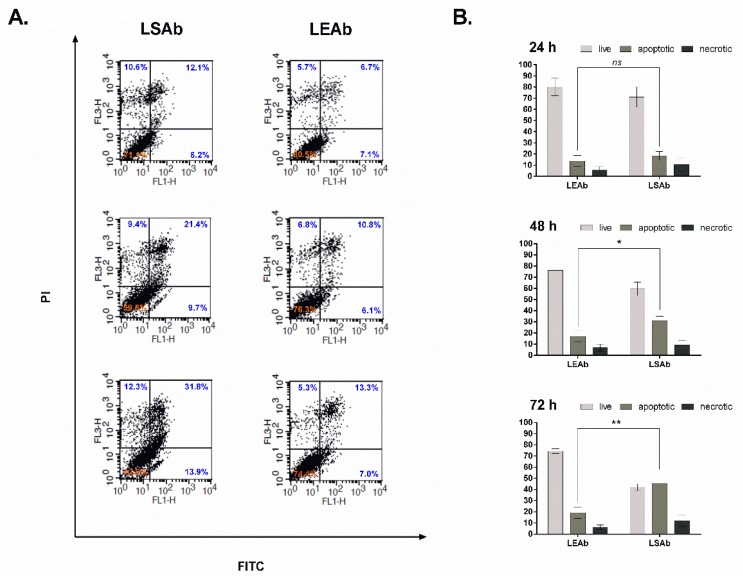
Determination of apoptosis/necrosis level after 24, 48 and 72 h of incubation with immunoliposomal form of simvastatin or control empty immunoliposomes (6.5 µM of simvastatin). SKBR3 cells were labelled with Annexin V-FITC and PI and analysed by flow cytometry. Quadrant legend: upper left—necrotic cells, upper right—cells in late apoptosis, lower left—viable cells, lower right—cells in early apoptosis. LSAb—immunoliposomes with simvastatin, LEAb—empty immunoliposomes. Statistical significance was determined on the basis of three independent experiments, by Student’s *t*-test, and differences were considered statistically significant at *p* < 0.05 (*), *p* < 0.005 (**), ns—not significant (*p* > 0.05). (**A**) Dot plots for SKBR3 cells treated with LSAb or LEAb; (**B**) Average number of live, apoptotic and necrotic cells after treatment with LSAb or LPAb. Data from three independent experiments.

To further demonstrate the effectiveness of targeted liposomes in vitro, the analysis of apoptosis induced in SKBR3 breast cancer cells treated with immunoliposomal simvastatin was performed. Cells were treated with immunoliposomal simvastatin or, as a control, empty immunoliposomes for 24, 48 and 72 h at a concentration of 6.5 µM simvastatin. To determine quantitatively the percentage of cells which undergo apoptosis or are necrotic, cells were stained with Annexin V-FITC and PI and analysed via flow cytometry. After 48 h immunoliposomal simvastatin induced apoptosis in almost 33% of analysed cells and, after 72 h, around 45% of cells were Annexin V positive (Figure 7), indicating that therapeutic activity of the proposed liposomes is based on apoptosis induction. Empty immunoliposomes used as a control, even after 72 h, did not induce apoptosis at a substantial level. More than 75% of treated cells were viable (Figure 7).

### 2.7. Immunoliposomal Form of Simvastatin Inhibits Signalling Pathways Involving Akt and Erk

**Figure 8:**
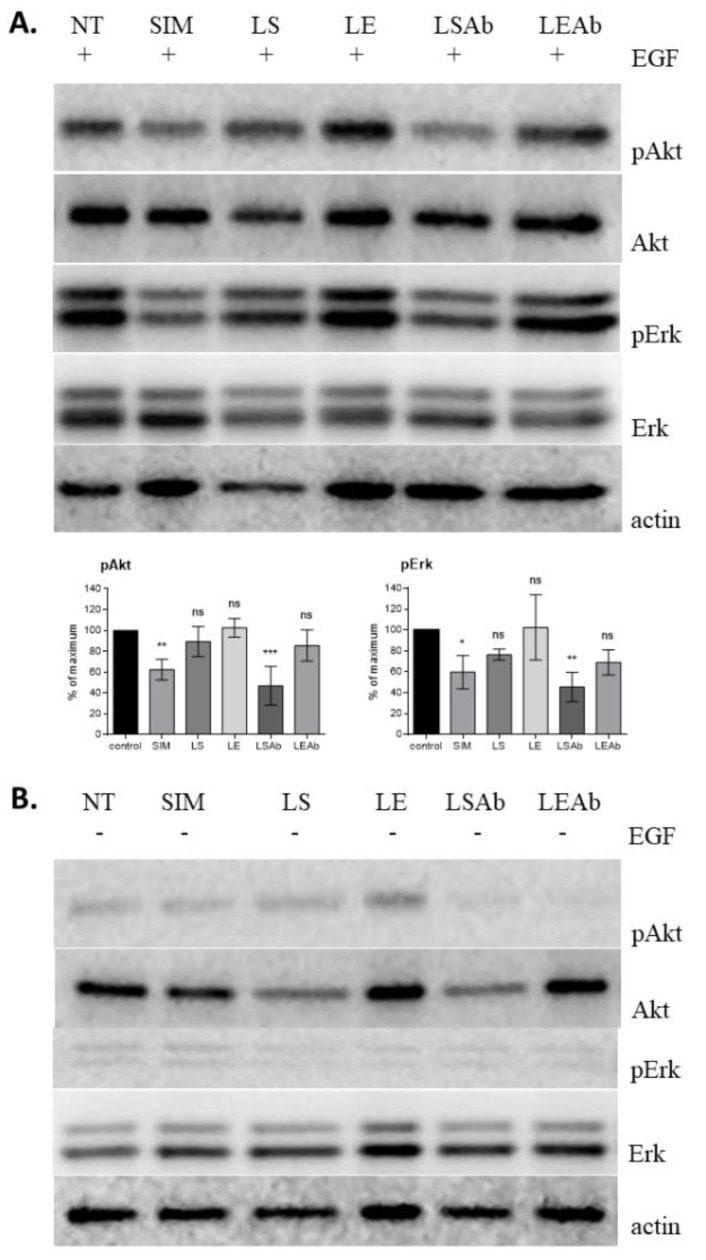
Levels of activation of Erk and Akt kinases in SKBR3 cells treated with simvastatin liposomes and simvastatin immunoliposomes. Cells were treated with liposomal forms of simvastatin for 24 h, and subsequently stimulated with EGF (100 ng/mL) for 15 min (**A**) or not stimulated (**B**), and then cell lysates were prepared and analysed by Western blot. NT: untreated cells; SIM: pure simvastatin; LS: non-targeted simvastatin liposomes; LE: non-targeted empty liposomes; LSAb: simvastatin immunoliposomes; LEAb: empty immunoliposomes. Statistical significance was determined on the basis of three independent experiments, by one way ANOVA with a post hoc Dunnett’s test, and differences were considered statistically significant at *p* < 0.05 (*), *p* < 0.005 (**), *p* < 0.0005 (***), ns: not significant (*p* > 0.05).

In the last part of the present study we analysed changes in intracellular signal transduction induced by liposomal forms of simvastatin. Considering that the PI3K/Akt/mTOR and MAPK/Erk signalling pathways are particularly important in cancer progression and both signalling pathways may be activated by signals transmitted via EGFR, we decided to check whether simvastatin present in its liposomal form would have an identical (or stronger) effect on cancer cells compared to free form of the drug presented before in many studies, e.g., [26]. Cells were treated at concentrations equal to IC_50_ with the immunoliposomal form of simvastatin and, to compare, with non-targeted simvastatin liposomes and the drug in its free form. After 24 h incubation, the phosphorylation level of Akt and Erk kinases was detected by Western blot analysis using appropriate antiphospho-ERK1/2 or antiphospho-Akt antibodies (Figure 8).

Simvastatin immunoliposomes reduced phosphorylation levels of both Akt and Erk kinases upon treatment with EGF. The effect of simvastatin immunoliposomes was probably the result of drug and antibody synergetic action coincidence, as empty immunoliposomes also slightly decreased the phosphorylation level of both kinases. Detailed statistical analysis was performed to confirm that the effect observed for simvastatin immunoliposomes is a result of the presence of a drug, not only antibodies. The differences in the level of inhibition of kinase phosphorylation by the free drug in comparison to untreated cells and simvastatin immunoliposomes in comparison to empty immunoliposomes are statistically significant, which proves that the result observed for simvastatin immunoliposomes is not solely the effect of antibodies. However, in the case of untargeted liposomes, the observed differences between the drug carrier and the empty carrier are not statistically significant for any of the tested kinases. It may suggest that the attachment of the targeting antibody to simvastatin liposomes facilitates cell-liposome interactions (Figure 8A).

These results may suggest that the presence of immunoliposomal simvastatin causes the largest decrease in phosphorylation level of Akt (PI3K/Akt/mTOR pathway) and Erk (MAPK/Erk pathway) kinases after 24 h of incubation.

## 3. Discussion

The idea of delivery of an encapsulated drug directly to selected cells using targeted liposomes is frequently used with rather promising outcomes. HER2-overexpressing cancer cells are a common target for immunoliposomes loaded with different agents, and many studies have shown that this type of treatment is very effective [23,27].

The concept of using statins, in particular hydrophobic statins—which seem to exert better anticancer effects than hydrophilic ones—in cancer treatment has been known since the 1990s. Since then, many in vitro experiments have been performed, followed by in vivo and even cohort studies (reviewed in [28]). However, because of their mostly lipophilic character, poor solubility and pharmacokinetics, statin delivery remains problematic. Recently it was shown that the liposomal form of simvastatin is much more effective than its free form in in vivo treatment of colon carcinoma [29] and in treatment of cancers showing presence of tumor-associated macrophages (TAM) in tumor tissue [16]. Therefore, we decided to design long-circulating, targeted liposomes with simvastatin, a hydrophobic statin which shows high potency in cohort studies, along with a commercially available antibody, trastuzumab (humanized anti-HER2/neu antibody), as a specific agent defining the target of the drug. Of note, our formulation could also be envisioned being used for other systems for which statins show potency and surface markers are known, such as EGFR. Herein, we chose trastuzumab and breast cancer cell lines which are known to overexpress HER2 (SKBR3 and BT474) and, moreover, these cancer cell lines showed the highest sensitivity to liposomal simvastatin treatment among all tested breast cancer cell lines of different molecular subtypes. Furthermore, it was shown before that another hydrophobic statin, fluvastatin, may potentiate the effect of trastuzumab [30]. To further explore the mechanism underlying the efficacy of our immunoliposomal simvastatin, we compared the phosphorylation levels of Akt and Erk kinases as well as the ability to induce apoptosis in cells treated with immunoliposomal, liposomal and free simvastatin.

While designing the liposomes, we tested several formulations that differed in terms of composition. As the main component of the liposomal bilayer we chose HSPC—a hydrogenated phospholipid derived from soybean with fully saturated acyl chains (a mixture of C16 and C18 acyl chain phosphatidylcholines). Fifteen mole percent of HSPC was replaced by DSPC, resulting in increased stability of liposomal formulation. HSPC and DSPC are used in pharmacy interchangeably. For example, currently on the market there are two liposomal formulations of doxorubicin which differ in terms of the main component: Doxil is prepared mainly from HSPC and Lipo-Dox is prepared from DSPC. It was shown that lipid nanoparticles with longer acyl chain lipids show greater stability [31]. Our observations showed that replacement of 15 mol % HSPC with DSPC resulted in a significant increase not only in the stability of our preparations, but also in the increase of the efficiency of encapsulation. Perhaps the presence of long-chain, saturated PC makes better environment for hydrophobic compounds like simvastatin. We have observed previously similar effect for coenzyme Q loading into PC liposomes [32]. Moreover, to overcome rapid clearance of liposomes by the mononuclear phagocyte system (MPS) and extend their circulation time, we enriched our formulation with DSPE-PEG, which is known to limit immunogenic and antigenic reactions.

Our hypothesis was that simvastatin would successfully replace cholesterol in the liposomal bilayer, so to promote drug encapsulation, we focused mainly on changes in cholesterol content. We observed that the lower the cholesterol content in the liposome bilayer was, the later crystals of precipitated simvastatin appeared in the liposome suspension, meaning that the lower leakage of the drug from liposomes and the higher stability of the liposomal formulation. Unfortunately, stability of liposomes did not coincide with encapsulation efficiency: we observed that at least a 0.5:1 chol:SIM molar ratio is necessary to obtain encapsulation efficacy of around 80 µg of simvastatin/mg of phospholipids.

After designing and determining the composition of the simvastatin nanocarriers with high efficiency in terms of simvastatin concentration and the lowest toxicity of the formulation not containing a drug, we characterized our liposomes in terms of their physical properties and their stability. Both liposomes and immunoliposomes did not exceed the diameter of 130 nm. The size of liposomes is a crucial physical parameter during liposome design. Although due to the enhanced permeation and retention (EPR) effect, molecules with the size even of around 500 nm may pass through the fenestrations in tumour vasculature endothelium and accumulate in tumours, our goal was to develop drug carriers with the size close to 100 nm. It is known that larger liposomes are characterized by better drug loading values. Indeed, when we compared our liposomal formulation (123 ± 3 nm) with those of others having e.g., significantly larger size (180 ± 20 nm) [18], we found that our liposomes had lower encapsulation efficiency. However, it was demonstrated that larger liposomes interact better with plasma proteins and have shorter half-lives in the circulation, as they are captured easily by the reticuloendothelial system (RES). Therefore, we decided to compromise the encapsulation efficacy in order to gain a longer circulation time and kept the diameter of the final immunoliposome not larger than 130 nm. Nanoparticles of a size comparable to our simvastatin carriers are highly desirable in treatment in vivo because of their efficient extravasation through the tumour leaky vessels and their ability to avoid capture by RES (reviewed by Bozzuto [33]). Furthermore, they are well tolerated by living organisms, as was shown for simvastatin liposomes with the size of around 100 nm inhibiting tumours in melanoma-bearing mice [16].

Further, we analysed the stability of designed liposomes and immunoliposomes with simvastatin stored as a suspension at 4 °C. Stability tests were multiparametric in order to better characterise both formulations. The measurements of changes in drug content, physicochemical properties of nanocarriers such as diameter, zeta potential and PDI values and therapeutic efficacy of tested liposomes were carried out every 30 days. Stability tests are the basis to determine the shelf-life of the potential product, and their implementation is particularly important if the product is considered in terms of potential therapeutic use. Our liposomal and immunoliposomal formulations were stable in diameter for at least 12 months, but the drug content (DL), being stable for 8 months, decreased after this time to 75% of the initial value and was stable up to 12 months. Nevertheless, 8 months-long shelf-life of a product seems to be sufficient.

We also checked the stability of our immunoliposomes in the presence of human plasma. Contrary to results obtained for liposomes incubated in 4 °C, after incubation of immunoliposomes with human plasma we noted an increase in the diameter of immunoliposomes. The observed increase could be caused by the corona effect [34]. However, because immunoliposomes showed only minor changes in diameter after 24 h of incubation with human plasma, we considered them as stable and appropriate for future in vivo studies. Next, we determined the zeta potential stability of our liposomes. It turned out that, although they are not composed of anionic lipids like 1,2-dipalmitoyl-phosphatidyl-glycerol (DPPG) or dimyristoyl-phosphatidyl-glycerol (DMPG), liposomes have a negative charge which changes towards more neutral after antibody attachment. It is known that strongly anionic liposomes are rarely used in in vivo treatment because of their lower stability in the circulation, rapid uptake by RES and induction of pseudo-allergies [33,35]. However, it was also shown that small amounts of negatively charged lipids stabilize neutral liposomes against an uptake mechanism dependent on aggregation, so all cases should be considered individually [35]. In the case of our liposomes, once again we very carefully analysed simvastatin immunoliposomes after 24 h incubation with human plasma, and we found no significant changes in their diameter or zeta potential, suggesting that despite the slightly negative charge, our immunoliposomes are expected to be well tolerated by the body after intravenous administration.

The last step was to determine the drug-to-lipid ratio (DL) in our liposomes. We found a DL value within the range 6.1–6.8, so typical for lipid carriers with the drug incorporated passively into liposome membrane. Generally, DL value in most existing nanodrug formulations does not exceed 10% [36]. We also compared our results with results obtained by other groups that focused on simvastatin liposomes prepared via the lipid film hydration method. Some of those liposomes had better drug loading values, but as mentioned above they were larger than ours [18]. Some already published formulations had a DL value comparable to ours [29], or even lower [15,16,19]. Despite lower DL values, it was found that liposomal statin exerted a great inhibitory effect both in vitro and in vivo on tumour cell growth, in that case, on melanoma cells [16]. Most importantly, none of those liposomal formulations were targeted directly at cancer cells by a specific ligand; all of them used an EPR effect or were directed towards tumor-associated macrophages.

We also verified the amount of antibodies attached to our immunoliposomes, which was around 41.5 ng of antibodies per 1 nmol of phospholipids, and compared it with other HER2-targeted liposomal preparations of different drugs known from the literature [9,23]. The amount of antibodies attached to our liposomes was even lower than that used in in vivo experiments on athymic nude balb/c mice with liposomes loaded with doxorubicin [9], so we assumed that our formulation may be considered suitable for use in in vivo studies.

Viability studies confirmed that the lowest IC_50_ values were obtained for HER2 overexpressing cells treated with the immunoliposomal form of simvastatin. It is in agreement with our hypothesis that liposomes containing simvastatin, targeted by antibodies attached to their surface, would be selectively delivered to cancer cells. The selectivity of our immunoliposomes was confirmed by two methods, flow cytometry and confocal microscopy, both clearly demonstrating that targeted liposomes bind specifically to HER2 overexpressing cells.

It should be noted that MDA MB 231 cells appeared to be most sensitive to free simvastatin among studied breast cancer cell lines and in vitro less sensitive to the liposomal form of the drug. High sensitivity of MDA MB 231 cells for simvastatin treatment may be correlated, among other factors, with expression status of EGFR. Other features known from the literature that may predispose cells to statin treatment are presented in Table 5. We compared determined IC_50_ values for breast and prostate cancers and we observed that for those cancer cell lines that overexpress EGFR (MDA MB 231, SKBR3, LNCaP and PC-3 (Matusewicz and Ligas, [24])) IC_50_ values for simvastatin were lower than 10 µM in comparison to cells without or with low EGFR expression (MCF7 and BT474) for which IC_50_ values were 50 µM or more. High expression of EGFR correlates with advanced stage of tumour development and poor response to chemotherapy. Studies of the metabolism of plasma cholesterol during tumour formation [37] revealed that in mice, after tumour formation, the plasma cholesterol level significantly decreases, suggesting that tumorigenesis itself requires higher cholesterol levels. Because MDA MB 231 and SKBR3 cell lines, derived from metastatic sites, represent fast-growing and tumor-forming breast cancer that do not overexpress the oestrogen receptor (ER-), they may be less sensitive to external cholesterol depletion than MCF7 and BT474 cell lines that represent tumours with a slower growth rate and ER+ status (see Table 5). The state after tumorigenesis is also correlated with statin sensitivity: a few cohort studies emphasize that statin use may exert a greater effect on cancer progression, but not initiation [38,39]. In addition, it was shown that the simvastatin-sensitive breast (MDA MB 231 and MCF7) and prostate (PC-3, LNCaP) cancer cells selected by us have higher cholesterol and lipid rafts/caveolae levels than their normal counterparts and were more sensitive to cholesterol depletion [40]. This supports our assumption that sensitivity to statins depends on the location of EGFR in membrane rafts. Disruption of the structure of such domains by cholesterol depletion may lead to modification of signal transduction. We hypothesize that simvastatin may specifically disorganize membrane rafts and therefore disturb the EGFR-dependent signalling pathways which usually promote cell proliferation and metastasis in epithelial cancers [41,42,43].

There is substantial evidence that membrane rafts play a very important role in cancer, including breast, prostate [48] and human liver cancer [49] development and progression reviewed in [41]. It was shown that EGFR present in membrane rafts promotes phosphorylation and activation of Akt [50,51]. Lack of EGFR phosphorylation in simvastatin-treated cells suppresses Akt and Erk activity. We demonstrated that in SKBR3 cells inhibition of both kinases by an immunoliposomal form of simvastatin is significant and similar to the free drug, as we expected. Targeting two or more signal transduction pathways is highly desired in therapeutic strategy; therefore, simvastatin delivered selectively to cancer cells seem to be very promising.

It was previously demonstrated that after statin treatment, almost all known molecular mechanisms of apoptosis become activated [28]. We found that our simvastatin immunoliposomes may also induce apoptosis in the SKBR3 cancer cell line at a level comparable to the free drug, reaching around 60% of apoptotic cells after 72 h of treatment, while treatment of the same cells with empty immunoliposomes led to mild apoptosis which remained at the same level (around 15%) during 72 h of incubation. This result confirms that our immunoliposomes may easily serve as a safe simvastatin delivery system which will not cause rapid cell death and should not induce inflammation.

Anticancer therapies with simvastatin, although promising, are currently hardly possible due to poor simvastatin bioavailability, its toxicity in high concentrations and the lack of simvastatin delivery systems. Here we propose using simvastatin immunoliposomes to deliver the drug directly to selected cancer cells. The model proposed here, which considers using a drug showing high potency towards EGFR overexpressing cells targeted against cells with high expression of the EGFR homologous receptor HER2, seems advantageous over other attempts to deliver relatively high quantities of statins to the target cells. Many studies have reported that cells treated only with trastuzumab may become resistant to this drug. In this case, cells overexpress EGFR and its strong signalling promotes cancer cell survival [52], in spite of trastuzumab binding to HER2 remaining unchanged [53]. It was previously reported that the use of Akt kinase inhibitor together with trastuzumab overcame trastuzumab resistance in BT474 and SKBR3 cell lines [54]. We believe that the use of HER-2 targeted liposomal simvastatin may become an equally effective solution.

The immunoliposomal formulation of simvastatin presented here is characterized by a favourable drug-to-lipid ratio, long-term stability, high selectivity and good anti-tumor potency in vitro. Additionally, it is also possible to attach to the liposomal envelope other antibodies specifically recognizing different targets, so the described formulation may be considered universal and its use is limited only by the differential sensitivity of cancer cells to statins. Considering the results of our in vitro studies, we believe that our liposomal formulation is a reasonable candidate for future evaluation of efficacy in vivo, when choosing the appropriate animal model. It is also of great importance to further study the molecular mechanisms triggered by statins in tumours, which has not been completely explained so far. These may answer the question about the legitimacy of using statins in monotherapy or in combination in anticancer therapies in the future.

## 4. Materials and Methods

### 4.1. Materials

Simvastatin was purchased from Cayman Chemical Company (Ann Arbor, MI, USA), hydrogenated soya phosphatidylcholine (Phospholipon 90H, HSPC) was purchased from Lipoid GmbH (Ludwigshafen, Germany), 1,2-distearoyl-sn-glycero-3-phosphocholine (DSPC) was purchased from Avanti Polar Lipids Inc. (Alabaster, AL, USA), cholesterol was purchased from Northern Lipids Inc. (Vancouver, BC, Canada), N-(carbonyl-methoxypolyethyleneglycol 2000)-1,2-distearoyl-sn-glycero-3-phosphoethanolamine, sodium salt (Sunbright DSPE-020CN, DSPE-PEG) and N-[(3-maleimide-1-oxopropyl)aminopropyl polyethyleneglycol-carbamyl] distearoylphosphatidyl-ethanolamine (Sunbright DSPE-020MA, DSPE-PEG-Mal) were purchased from NOF Corporation (Shibuya, Tokyo, Japan). The antibody Herceptin (trastuzumab) was purchased from Roche (Roche Registration Limited, Welwyn Garden City, UK). Further reagents were: 2-iminothiolane hydrochloride (Traut’s reagent), Sepharose 4B, propidium iodide, Bicinchoninic Acid Kit for Protein Determination (BCA) and thiazolyl blue tetrazolium bromide (MTT) which were purchased from Sigma-Aldrich (St Louis, MO, USA), Accutase Cell Detachment Solution which was purchased from Corning (Manassas, VA, USA), Vybrant DiD Cell-Labeling Solution which was purchased from Thermo Fisher Scientific (USA), primary antibodies: HER2/ErbB2 (#2242; 1:1000), EGFR/ErbB1/HER1 (#2232; 1:1000), phosphorylated Akt (Ser473; #4060; 1:1000), Erk (#4696; 1:1000), phosphorylated Erk (Thr202/Tyr204; #4370; 1:1000) were from Cell Signaling Technology (Beverly, MA, USA), Akt1 (#sc-5298; 1:1000), and actin (#sc-1616; 1:1000) and HRP-conjugated appropriate secondary antibodies were from Santa Cruz Biotechnology (Santa Cruz, CA, USA).

### 4.2. Preparation of Non-Targeted Liposomes with Simvastatin

Long circulating liposomes with simvastatin incorporated into the liposome bilayer were prepared by lipid film hydration. Briefly, HSPC, DSPC, cholesterol, DSPE-PEG and DSPE-PEG-Mal dissolved in chloroform were mixed with simvastatin dissolved in methanol in a molar ratio of 4.37:1:0.3:0.3:0.05:0.63. Lipids and drug were dried under a stream of nitrogen and kept under vacuum overnight to completely remove solvents. The lipid film was rehydrated for 20 min at 64 °C with Modified Earle’s buffer solution/4-(2-hydroxyethyl)-1-piperazineethanesulfonic acid (HEPES) and 2-(N-morpholino) ethanesulfonic acid (MES) buffer pH 7.2 (50 mM MES, 50 mM HEPES, 75 mM NaCl). Liposome size was reduced by at least 10 extrusion steps sequentially through polycarbonate membranes with 400, 200 and 100 nm pores (Nucleopore, Whatman, Sigma-Aldrich, St. Louis, MO, USA). The free drug was removed by gel filtration via size-exclusion chromatography on a Sepharose 4B column equilibrated with HEPES buffer.

### 4.3. Antibody Conjugation to Liposomes

To obtain targeted, long-circulating, therapeutic liposomes (immunoliposomes) containing simvastatin, maleimide-PEG-DSPE at 0.8 mol % of the coat formulation was included. In order to attach trastuzumab (Herceptin) to the liposomal surface, free amino groups of the antibodies were thiolated by exposure to 2-iminothiolane (Traut’s reagent) for 4 h at 4 °C in HEPES buffer. The Traut’s reagent was removed by overnight dialysis into HEPES buffer and -SH groups of the antibody pre-formed at the previous step reacted with maleimide groups on the surface of liposomes during incubation for up to 24 h; the molar ratio of DSPE-PEG-Mal in the liposomal bilayer to antibody was adjusted to 13.3:1 [22]. Unbound antibodies were removed by gel filtration via size-exclusion chromatography on a Sepharose 4B column equilibrated with HEPES.

The density of trastuzumab on the surface of immunoliposomes was measured using the sandwich ELISA. In brief, a 96-well plate (Greiner Bio-One GmbH, Kremsmünster, Austria) was exposed to 0.4 μg of polyclonal rabbit anti-human IgG (Jackson ImmunoResearch, Cambridgeshire, UK) in 100 μL of PBS per well. The plate was incubated overnight at 4 °C, washed with PBS and blocked with 5% non-fat milk in PBS for 1 h at 37 °C. Next, the plate was washed and immunoliposomes with simvastatin or empty immunoliposomes at a dilution of 1:5000 or 1:10,000 were added and incubated for 1 h at 37 °C. Subsequently, the plate was washed again and incubated for 45 min with a secondary goat antihuman IgG conjugated with HRP (Abcam, Cambridge, MA, USA), at a dilution of 1:10,000. After further washing with PBS, activity of HRP was detected with the TMB Substrate Kit (Thermo Scientific, Waltham, MA, USA). The reaction was stopped by addition of 100 µL of 2 M sulfuric acid and the results were read on a multi-well plate reader (Rayto, Shenzhen, China) at wavelength (λ) = 450 nm.

### 4.4. Particle Size and Zeta Potential Analyses 

Mean particle size, polydispersity index (PDI) and zeta potential values of the non-targeted liposomes and immunoliposomes were determined by dynamic light scattering (DLS) using the Zetasizer Nano ZS (Malvern Instruments Ltd., Malvern, UK).

### 4.5. Determination of Drug Encapsulation Efficiency and Drug Loading

The amount of simvastatin incorporated into the liposomal bilayer was measured according to spectrophotometric method, which gives results comparable with RP-HPLC [55]. Briefly, 10 µL of simvastatin non-targeted liposomes or simvastatin immunoliposomes were solubilized with 1 µL of 10% SDS. Samples were vortexed and incubated at room temperature for 2 min. Next, 1 mL of methanol was added and simvastatin content was measured using an UV-VIS spectrophotometer (Cary 50 Bio UV-Visible Spectrophotometer, Varian, Palo Alto, CA, USA) at λ = 238 nm. The concentration of simvastatin was determined from the absorption coefficient 24,781.12 L·mol^−1^·cm^−1^ and the calibration curve in methanol. Total lipid content of the liposomal dispersion was determined via a phosphate assay according to Rouser et al. [56].

Encapsulation efficiency (EE) was calculated as:

EE (%) = (SIM in liposomes (mg)/SIM initially added (mg)) × 100(1)

Drug loading (DL) was calculated as:

DL (%) = (mg of SIM in liposomes/mg of phospholipids in liposomes) × 100(2)

SIM—simvastatin

### 4.6. Stability of Simvastatin Immunoliposomes

Non-targeted simvastatin liposomes, simvastatin immunoliposomes and their empty counterparts were stored at 4 °C for 1 year. After every 30 days aliquots from all liposomal formulations were taken and their size and zeta potential were measured. Additionally, aliquots of immunoliposomes and non-targeted liposomes with simvastatin were placed on Sepharose 4B mini-columns (10 × 0.7 cm) to separate simvastatin which might have been released from liposomes during storage, the most concentrated fractions were collected and the drug-to-phospholipids ratio of both kinds of liposomal formulations was calculated as described above for the determination of DL (%).

To determine the stability of immunoliposomes in the presence of human plasma, recently outdated human peripheral blood was collected from the local blood bank and subsequently plasma was separated from the red blood cells by centrifugation at 800 × g for 10 min at 4 °C. Equal amounts of immunoliposomes and human plasma were mixed and incubated for up to 24 h at 37 °C. After 1, 2, 4, 6 and 24 h aliquots of 200 µL were taken, placed onto Sepharose 4B mini-columns (10 × 0.7 cm), the most concentrated immunoliposomal fractions were collected, and the size and zeta potential of immunoliposomes were measured.

### 4.7. Cell Culture

The human breast and prostate cancer cell lines SKBR3, MDA MB 231, BT474, MCF7 and PC-3 were obtained from the American Type Culture Collection (ATCC, Manassas, VA, USA). SKBR3 cells were cultured in McCoy’s 5A modified medium supplemented with 10% FBS, 2 mM L-glutamine and 1% penicillin-streptomycin. MDA MB 231 and PC-3 cells were cultured in Dulbecco’s modified Eagle’s medium (DMEM) supplemented with 10% FBS, 2 mM L-glutamine and 1% penicillin-streptomycin. BT474 cells were cultured in RPMI 1640 medium supplemented with 10% FBS, 2 mM L-glutamine, 1% penicillin-streptomycin and 20 μg/mL insulin. MCF7 cells were cultured in Eagle’s minimum essential medium (EMEM) supplemented with 10% FBS, 2 mM L-glutamine and 1% penicillin-streptomycin. Cells were maintained in a humidified 5% CO_2_ air atmosphere at 37 °C.

### 4.8. Selectivity of Immunoliposomes towards Breast Cancer Cells Overexpressing HER2

Detection of immunoliposomes interacting with cells was determined by flow cytometry and confocal laser scanning microscopy.

For flow cytometry analysis, breast cancer cells were suspended at a density of 1 × 10^5^ cells in 500 µL of fresh medium. Cells were treated with DiD-labelled immunoliposomes or non-targeted liposomes at 30 µM lipid concentration and incubated for 30 min or 2 h. After each time interval, cells were analysed with a BD FACSCalibur flow cytometer (BD Biosciences, Singapore) by collecting 10,000 events for each sample in the FL-4 channel. Untreated cells were used as a negative control. Life-gating was performed by using forward vs side scatter to exclude debris and dead cells. The gating was performed separately for each cell population.

For confocal laser scanning microscopy MDA MB 231 and BT474 breast cancer cells were seeded at 7.5 × 10^4^ cells/well on cover slips in 24-well plates and incubated for 24 h. The next day, cells were treated with DiD-stained immunoliposomes and non-targeted liposomes at 30 μM lipid concentration and incubated for 2 h at 37 °C. After that, cells were washed three times with cold PBS to remove unbound liposomes, fixed for 20 min with 4% paraformaldehyde (PFA) and mounted on a cover glass with Fluoroshield/DAPI histology mounting medium (Sigma-Aldrich, St Louis, MO, USA). Images were captured on a Zeiss LSM 880 laser scanning microscope with Airyscan (Carl ZEISS, Jena, Germany). Samples were excited at 405–430 nm for DAPI and 633 nm for DiD and imaged with a Plan-Apochromat 63×/1.4 Oil objective, using 415–543 nm and 642–695 nm filters for DAPI and DiD respectively.

### 4.9. Cellular Viability Assay

All tested cell lines were seeded at 5000 cells/well in 96-well plates and incubated for 24 h. Simvastatin was dissolved in ethanol and then diluted in cell culture medium as required. The dilution of simvastatin in medium for the highest concentration used in experiments was 1:250, which excludes the toxic effect of ethanol itself. Cells were treated with free simvastatin, non-targeted empty liposomes, non-targeted liposomes with simvastatin, immunoliposomes with simvastatin and empty immunoliposomes, in complete cell culture medium. After 48 h incubation, cell viability was determined using the MTT assay, according to the standard protocol. Absorbance was measured using a Rayto microplate reader at λ = 492 nm. The IC_50_ value was determined from the concentration-effect curve, in which the viability of the control cells was considered as 100%.

### 4.10. Determination of Apoptosis 

Cells (2.5 × 10^5^ cells) were seeded on 12-well plates, incubated for 24 h and then treated with both simvastatin-containing and empty non-targeted liposomes or immunoliposomes for 24, 48 and 72 h. At indicated time points, cells were detached with Accutase, harvested, washed twice with cold PBS and then suspended in a binding buffer. Cells were stained with FITC Annexin V and propidium iodide (PI) for 15 min, according to the manufacturer’s protocol (FITC Annexin V Apoptosis Detection Kit I, BD Pharmingen, Singapore), and analysed using a BD FACSCalibur (BD Biosciences, Singapore) for FITC and PI fluorescence based on 10,000 counts for each population.

### 4.11. Signalling of Pathways Involving Akt and Erk

Cells (2.5 × 10^5^) were cultured on 12-well plates in medium without FBS for up to 16 h, then medium was refreshed and cells were treated with immunoliposomes and non-targeted liposomes with simvastatin. After 24 h incubation, cells were stimulated by 100 ng/mL EGF (EGF Human Recombinant, Pichia, ProSpec, Rehovot, Israel) for 15 min. Cell lysates were prepared and activation levels of Akt and Erk were visualised by Western blot analysis. Enzymatic reaction was developed by using the ECL procedure with luminol (Sigma-Aldrich, St Louis, MO, USA) and coumaric acid (Sigma-Aldrich, St Louis, MO, USA USA) as substrates and chemiluminescence readout. Scanning was performed using UVP Chemi HR410. The intensity of the signal was quantified by densitometric analysis using ImageJ software (software version, 1.52e, University of Wisconsin, WI, USA).

### 4.12. Statistical Analyses

All experiments performed were replicated at least three times. For the results shown in Figure 4 and Figure 6, Figure 7, Figure 8 a representative example is shown. For viability experiments and for experiments where activity of kinases was analysed, the results obtained from non-treated cells were considered as 100% values, to which all other values were compared. The data are presented as means ± standard error.

All statistical analyses were performed using GraphPad Prism (software version 6.01, La Jolla, CA, USA). Differences between groups were tested by Student’s *t*-test, or one way ANOVA as indicated in figure legends and a *p* value < 0.05 was considered statistically significant.

## 5. Conclusions

In conclusion, our study presents novel, immunoliposomal form of simvastatin, characterised by high stability, selectivity and in vitro effectiveness. Furthermore, we point at cells overexpressing EGFR as particularly sensitive for simvastatin treatment. Our findings give strong basis for further studies on targeted formulations of statins in cancer therapy.

## Figures and Tables

**Table 1:** Studied liposomal formulations.

Formulation No.	Formulation with mol %	Chol:SIM Molar Ratio	Stability (days)	EE (%)
1	HSPC:DSPE-PEG:chol:SIM 8.0:0.5:1.0:1.0	1:1	30	50.3
2	HSPC:DSPE-PEG:SIM 8.5:0.6:1.5	0:1	45	40.8
3	HSPC:DSPE-PEG:chol:SIM 8.5:0.6:0.5:1.0	0.5:1	40	51.3
4	HSPC:DSPC:DSPE-PEG:chol:SIM 6.9:1.6:0.5:0.5:1.0	0.5:1	240 *	67.8

* after 8 months 85% of the initial amount of SIM was determined in immunoliposomes (see Figure 2). EE: encapsulation efficiency; HSPC: hydrogenated soya bean phosphatidylcholine; DSPE-PEG: 1,2-distearoyl-sn-glycero-phosphoethanolamine-N-(poly [ethylene glycol] 2000); chol: cholesterol; SIM: simvastatin; DSPC: 1,2-distearoyl-sn-glycero-3-phosphocholine.

**Table 2:** Lipid compositions of tested empty liposomes.

Lipid Composition of Empty Liposomes No. 1		Lipid Composition of Empty Liposomes No. 2	
**Component**	**Content (mol %)**	**Component**	**Content (mol %)**
HSPC	75.15	HSPC	65.65
DSPC	15	DSPC	15
DSPE-Peg	5.35	DSPE-Peg	5.35
**Cholesterol**	**4.5**	**Cholesterol**	**14**

**Table 3:** Properties of liposome formulations. Average size, polydispersity index (PDI) and zeta potential of particles.

Formulation	Size (nm)	PDI	Zeta Potential (mV)	EE (%)	DL (%)
Non-targeted empty liposomes	122.9 ± 1.2	0.046 ± 0.010	−16.7 ± 1.2	-	-
Non-targeted liposomes containing simvastatin	117.3 ± 1.6	0.037 ± 0.011	−17.6 ± 3.7	63.3 ± 12.4	6.8 ± 1.1
Empty immunoliposomes	127.6 ± 2.9	0.039 ± 0.014	−9.9 ± 2.7	-	-
Immunoliposomes containing simvastatin	123.1 ± 3.1	0.054 ± 0.015	−11.1 ± 3.5	64.6 ± 9.7	6.8 ± 1.4

DL: drug loading.

**Table 4:** IC_50_ values for inhibition of cellular viability in breast cancer cell lines after 48 h treatment with free simvastatin and its liposomal and immunoliposomal form.

Caption	MDA MB 231	MCF7	BT474	SKBR3
Simvastatin	2.3 µM	56.2 µM	194 µM	6.8 µM
Liposomal simvastatin	8.6 µM	44.6 µM	76.9 µM	7.1 µM
Immunoliposomal simvastatin	6.9 µM	49.8 µM	25.2 µM	6.5 µM

**Table 5:** Comparison of cells that are sensitive or less sensitive to cholesterol depletion.

Feature	MDA MB 231 and SKBR3	MCF7 and BT474
External cholesterol requirement (see Figure 1)	low	high
Susceptibility to statin treatment [2,44], (see Table 4)	high	low
ER status [1]	negative	positive
Tumour growth rate [45]	high	low
Expression of “signature” genes, among others HMGCR [46].	lower	Higher *
Transcription of genes involved in cholesterol synthesis after statin treatment	stays at the same level (lower levels of cholesterol required to survive)	increase (higher levels of cholesterol required to survive **)
Derived from	metastatic site	metastatic site/mammary gland
EGFR expression [1]	high	very low

* Only data for estrogen receptor (ER)-positive breast cancers reached statistical significance, suggesting that low expression of cholesterol biosynthesis genes is associated with longer recurrence-free and overall survival and may predict sensitivity to statin treatment. ** e.g., a primary metabolite of cholesterol, 27-hydroxycholesterol, may serve as an ER ligand and increase tumour growth in mice bearing ER+ breast cancer tumours [47].
